# Variation in Child Serum Cholesterol and Prevalence of Familiar Hypercholesterolemia: The Health Oriented Pedagogical Project (HOPP)

**DOI:** 10.1177/2333794X221079558

**Published:** 2022-03-23

**Authors:** Ester Fabiani, Martin Frank Strand, Morten Lindberg, Nandu Goswami, Per Morten Fredriksen

**Affiliations:** 1 University Medical Centre Ljubljana, Slovenia; 2 Alma Mater Europaea-ECM Maribor, Maribor, Slovenia; 3 Kristiania University College, Oslo, Norway; 4 Vestfold Hospital Trust, Tonsberg, Vestfold, Norway; 5 Medical University of Graz, Graz, Steiermark, Austria

**Keywords:** cholesterol, children, familiar hypercholesterolemia, prevalence, hyperlipidemia

## Abstract

Early stages of atherosclerosis may develop in childhood due to hyperlipidemia. The aims are to investigate the prevalence of familiar hypercholesterolemia in 6 to 12-year-old children and to study the deviation in cholesterol measures. Anthropometric data and venous blood were collected from children participating in the Health Oriented Pedagogical Project (HOPP). Out of 18 children with TC > 6.0 mmol/L, 15 were tested genetically and none diagnosed with FH. The prevalence of TC > 6.0 mmol/L declined from 1.3% in 2015 to 0.5% in 2016. The mean TC was 4.30 mmol/L both years, which is lower than in earlier studies. Usage of a single TC measurement and a threshold of TC > 6.0 mmol/L in screening children for FH, may not be a good screening strategy. While lipid values have a good reliability across 2 measurements, there are variations in individual TC levels across 1 year.

**What’s known on this subject?**
Atherosclerotic plaques are known to start in childhood due to high levels of cholesterol. Therefor young adults may be affected by coronary artery disease. Children with known risk factors, such as genetic predisposition, including familial hyperlipidemias, diabetes, and renal diseases, are at higher risk. With childhood obesity becoming increasingly more common this cholesterol levels are further highlighted as an important issue affecting children’s health. There are unclear recommendations regarding cholesterol screening in children and when to initiate hyperlipidemia treatment. It is of importance that measurements of cholesterol in children are reliable as to refer to further follow-up at a specialist.
**What this study adds?**
The present study adds the current state of cholesterol level in children living in a developed country. In addition, the reliability of repeated measures as well as the cut-off value as a mean to screen children for familiar hypercholesterolemia are questioned.

## Introduction

Hypercholesterolemia is a risk factor for atherosclerosis and cardiovascular diseases (CVD) in adults.^1^ Several studies have established that atherosclerosis may start to develop already in childhood, with deposits detectable from early ages.^2^ High serum cholesterol, high levels of low-density lipoproteins (LDL) and overweight may lead to increased risk of CVD, disability and death.^3^ Several studies display findings of overweight and obesity^4^ high serum cholesterol levels^5^ and several studies have linked child cholesterol levels with risk of CVD in adults.^6^ Hypercholesterolemia is mainly caused by lifestyle related factors as overweight, smoking, lack of physical activity or a combination of the above and are treated using statins.

In addition, the prevalence of genetic familiar hypercholesterolemia (FH) in the population is approximately 1 in 200 to 300 individuals.^7^ Routine screening in children has been suggested^8^ but there are no generally accepted screening programs for FH in children and adolescents.^9^ Most children diagnosed with FH are diagnosed through family cascade screening.^10^ Statin use in Norwegian children is increasing and higher in Norway than other scandianvian countries.^10^ To optimize screening strategies, its important to evaluate the effectiveness of high serum cholesterol as a predictor of FH in children. In addition, more knowledge on how serum lipids vary over time in healthy children is needed.

The aims of this study are to investigate the prevalence of hypercholesterolemia and FH as well as to investigate measurement variation in serum lipids levels, in healthy 6 to 12-year-old children.

## Methods

The data in this study was collected from the Health Oriented Pedagogical Project (HOPP)—a longitudinal controlled school-based physical activity intervention program.^11^ The Regional Committees for Medical and Health Research Ethics approved the study protocol (2014/2064/REK sør-øst) on 9 January 2015. The study is registered as a clinical trial (ClinicalTrials.gov Identifier: NCT02495714). All children included in the study provided written parental consent.

### Data Collection

Children aged 6 to 12 years old from 9 elementary schools in the south-east part of Norway were invited to participate. Venous blood and anthropometric data were collected, and children with a recent infection were excluded. In 2015, 2271 (81% of population) were tested, with 1340 (48%) consenting to blood sampling. In 2016, 2109 (75% of population) were tested, with 1167 (42%) consenting to blood sampling. In total 1051 sampled blood both years. Most children were Caucasian, and age of each child was defined at the day of venepuncture. Blood samples were collected in the non-fasting state between 8:00 a.m. and 1:30 p.m., and strenuous exercise prior to collection was avoided. Total cholesterol (TC) and high-density lipoprotein (HDL) cholesterol were analyzed on Vitros 5.1 (Ortho-Clinical Diagnostics, USA) with reagents from the supplier. Non-HDL cholesterol (nonHDL) was calculated as nonHDL = TC − HDL. Children with a TC > 6.0 mmol/L in 2015 were invited to test genetically for FH.

### Hypercholesrerolemia and FH

Hypercholesterolemia was defined as total cholesterol (TC) ≥5.2 mmol/L^12,13^ alone or in combination with HDL <1.0 mmol/L and/or nonHDL ≥3.8 mmol/L.^13^ Possible familiar hypercholesterolemia was defined as TC ≥6.0 according to the Norwegian criteria for FH screening. To provide additional predictive power the TC/HDL (Castelli Risk Index one) and nonHDL/HDL ratio (Atherogenic coefficient) were chosen. Variables of children’s overweight and obesity were based on Cole et al^14^ isoBMI scale.

### Statistics

Statistical analyses were completed with the IBM SPSS v. 23 (IBM, Armonk, NJ, USA) and R (version 3.5.1). Differences between 2015 and 2016 were calculated using appropriate hypothesis testing with ANOVA, depending on normality and type of variables. Kolmogorov Smirnov test was used to test normality. For non-normal distributed variables Mann-Whitney- and Kruskall Wallis tests where used. The α-level was defined at 0.05. Intraclass correlation coefficients (ICC) including 95% confident intervals were calculated for “single raters absolute” using the ICC function in the psych package in R.^15^ Quartile ranges and Pearson correlation was analyzed using R, and plots were generated using ggplot2 and ggstatsplot.

## Results

### Sample

Anthropometric variables are shown in Table 1. The mean height and weight increased with age, in addition to a significant difference in isoBMI (*P* < .001), waist circumference (WC) (*P* < .001), and Waist-to-height ratio (*P* < .001).

**Table 1.** Anthropometric Variables With *P*-Values for Test-Year.

	2015	2016	*P*-value*
	n = 2271	n = 2109	
Height (cm)	138.5 (11.8)	144.3 (11.9)	<.001
Weight (kg)	33.3 (9.6)	38.3 (10.8)	<.001
isoBMI	17 (3)	18 (3)	<.001
WC (cm)	63.0 (8.1)	65.0 (8.7)	<.001
WtHR	0.46 (0.05)	0.45 (0.07)	<.001

Data are mean (SD).

Abbreviations: IsoBMI, iso Body Mass Index; WC, waist circumference; WtHR, waist-to-height ratio.

* Mann-Whitney Test by year.

### Blood Lipids

The mean TC was 4.30 mmol/L both years (Table 2), and the HDL level was in the range of 1.63 to 1.67 mmol/L. Non-HDL level ranged 2.63 to 2.86 mmol/L and the TC/HDL ratio 2.67 to 2.76. There were no significant changes in TC or nonHDL between 2015 and 2016. However, there was an increase for HDL (*P* = .003), a decrease for TC/HDL (*P* = .001) and nonHDL/HDL (*P* = .001). Intraclass correlation coefficients (ICC) for all the variables were in the range of 0.78 to 0.82, indicating good reliability between years.

**Table 2.** Cholesterol Fractions Values With Intraclass Correlation Coefficient (ICC).

	2015	2016	*P*-value*	ICC (95% CI)**
	n = 1340	n = 1167		
TC (mmo/L)	4.30 (0.65)	4.30 (0.64)	.817	0.78 (0.76-0.80)
HDL (mmol/L)	1.63 (0.35)	1.67 (0.36)	.003	0.80 (0.78-0.81)
nonHDL (mmol/L)	2.68 (0.66)	2.63 (0.63)	.08	0.82 (0.81-0.84)
TC/HDL	2.76 (0.68)	2.67 (0.63)	<.001	0.82 (0.80-0.84)
nonHDL/HDL	1.76 (0.6)	1.67 (0.63)	<.001	0.82 (0.80-0.84)

Data are mean (SD).

* Mann-Whitney Test by year.

** ICC was calculated for the children who were measured both in 2015 and in 2016 (n = 1051).

The change in quartile distribution for TC and HDL across 2015 and 2016 is shown in Table 3. While there is no clear tendency for change in TC, the number of HDL values in Q3 and Q4 is increased, consistent with the significant increase from 2015 to 2016.

**Table 3.** Quartile Distribution of Total Cholesterol for Year 2015 and 2016.

		2015	2016	
		n	n	% change
Q1	TC	269	252	−6.3
	HDL	226	201	−11.1
Q2	TC	231	264	14.3
	HDL	228	216	−5.3
Q3	TC	269	243	−9.7
	HDL	323	327	1.2
Q4	TC	282	292	3.5
	HDL	274	307	12

Quartile distribution was calculated for the children who were measured both in 2015 and in 2016 (n = 1051), using quartiles defined from the 2015 data.

### Prevalence of Hypercholesterolemia and FH

The criteria for hypercholesterolemia,^13^ as well as Norwegian criteria for FH screening are presented in Table 4 along with prevalence for 2015 and 2016. For hypercholesterolemia, 9.6% of the children had TC ≥ 5.2 mmol/L in 2015, and 8.7% in 2016, with no statistically significant difference. The prevalence of children with both non-HDL ≥ 3.8 mmol/L and HDL < 1.0 mmol/L was 94 (7.0%) in 2015 and 56 (4.8%) in 2016. With Norwegian criteria for FH (Table 4), a significant decrease in prevalence was found between 2015 and 2016 (*P* = .03), from 1.3% in 2015 to 0.5% in 2016. In 2015 there were 18 children with TC > 6.0 mmol/L, matching the Norwegian criteria for screening for FH. Of these, 15 were tested genetically and none was diagnosed with FH.

**Table 4.** Prevalence of Hypercholesterolemia and Familiar Hypercholesterolemia.

		2015	2016
		n = 1340	n = 1167
Hypercholesterolemia criteria Uptodate^27^	(1) TC ≥ 5.2 mmol/L	129 (9.6)	101 (8.7)
	(2) HDL < 1 mmol/L and/or nonHDL ≥ 3.8 mmol/L	94 (7.0)	56 (4.8)
	Both (1) and (2) combined	65 (4.9)	0 (0)
Familiar hypercholesterolemia screening criteria for children (<20 years) in Norway^28^	TC ≥ 6.0 mmol/L	18 (1.3)	6 (0.5)

Data are in (%).

### Individual Variation

In 2016, 3 of the 18 children with TC > 6.0 mmol/L still had a value of TC > 6.0 mmol/L, 4 had decreased to the range of 5.2 to 6.0 mmol/L, and 2 had decreased below 5.2 mmol/L. Out of the 18, 9 opted out on the 2016 blood sample collection. In addition, 3 new children showed a TC > 6.0 mmol/L in 2016. In 2015, 66 children had TC between 5.2 and 6.0 mmol/L, while 30 remained in this category in 2016. Three increased to > 6.0 mmol/L, and 33 decreased to <5.2 mmol/L. A total of 79 (7.5%) children change TC category (above 6.0, between 6.0 and 5.2, and below 5.2 mmol/L). In addition, 31 children (2.9%) have a change of more than 1 mmol/L in TC (up/down) and 292 (27.8%) children have a change of more than 0,5 mmol/L in TC (up/down).

### Blood Lipids Over Time

**Figure 1.**
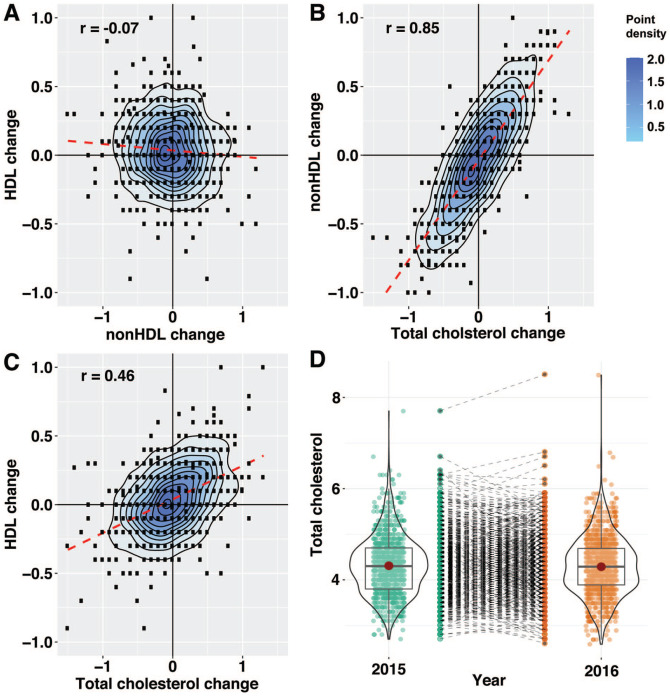
(A-C), scatterplots including density plots and correlation coefficients for lipid change 2015-2016. (A) nonHDL change versus TC change. (B) HDL change versus TC change. (C) HDL change versus nonHDL change. (D) Violin plot of TC for 2015 and 2016, with tracelines for each value in the middle. For (A-D) only values from children measured both years were included (n = 1051).

Scatterplots, with a density scale and with correlation coefficients, are shown in Figure 1 for combinations of the 3 parameters, HDL change, nonHDL change and TC change. In addition, a violin plot of TC for 2015 and 2016 is shown, including tracelines for TC change. Change in nonHDL cholesterol correlated strongly with total cholesterol change (*r* = .85, 95% CI (0.84, 0.87), *P* < .001) (Figure 1). Change in HDL cholesterol correlated with TC (*r* = .46, 95% CI (0.41, 0.50), *P* < .001), while change in HDL cholesterol had a weak negative correlation with nonHDL cholesterol (*r* = −.07, 95% CI (−0.13, −0.01), *P* = .019). In conclusion, most of the variation in TC is explained by variation in nonHDL, with some of the variation being explained by variation in HDL. There is a weak negative correlation between nonHDL and HDL.

## Discussion

Average serum TC, HDL, and non-HDL values from 2 subsequent years for a large pediatric population in Norway are presented. Also, changes in prevalence of hypercholesterolemia over 1 year are shown. Most of the variation in TC is explained by variation in nonHDL. The total variation in the population was low, but a considerable change in some individuals was observed. Out of 18 children with high TC in 2015, 15 were tested genetically, and none displayed FH.

### Lipid Levels

The mean TC was 4.30 mmol/L both years (Table 3), which is higher than from an Indian study (4.10 mmol/L for boys, 4.16 mmol/L for girls)^16^ but lower than from a Spanish study (4.2 mmol/L).^17^ The HDL level was higher in our population (1.63-1.67 mmol/L) than reported from Spain (1.3 mmol/L; LDL = 2.6 mmol/l, VLDL = 0.33 mmol/L.^17^ The nonHDL level was also lower in our study (2.63-2.86 mmol/L) as compared to data from Spain (2.9 mmol/L), but higher than those reported from a Danish study (2.3-2.4 mmol/L).^18^ The TC/HDL ratio was in the range of 2.67 to 2.76. While TC and nonHDL did not change from 2015 to 2016, HDL increased with 0.04 mmol/L, resulting in a reduction in the TC/HDL ratio. Although the increase in HDL was a statistically significant finding, the magnitude is not clinically significant at the individual level. However, increased HDL, modified by genetic predisposition, physical activity, and diet may reduce the amount of plaque in blood vessels.^19^ The elevated HDL-values in the present study may imply a healthier lifestyle.^20^ A lowered TC/HDL ratio in childhood may potentially reduce the risk of developing atherosclerosis in adulthood.^21^

### Prevalence of Hypercholesterolemia and FH

The prevalence of children with hypercholesterolemia was 9.6% (TC ≥ 5.2 mmol/L) in 2015 and 8.7% in 2016, compared to 3.3% in Denmark (TC > 5.2 mmol/L)^18^ and 7.8% in Germany.^22^ Studies in the US have reported a prevalence of high TC from 7.4% in the US NHANES study^19^ to 13% in fifth grade students in Arizona.^23^ The prevalence of low HDL decreased from 3.1% in 2015 to 2.5% in 2016. The reported prevalences of low HDL are 8.8% in Germany and >20% in France, Spain, and the Netherlands, while it is estimated to be >20% in Italy and Turkey.^24^ In Norway, national regulations state that values of TC ≥ 6.0 mmol/L and LDL > 3.5 mmol/L in children indicate a requirement of FH testing. While 18 children (1.3%) had a TC ≥ 6,0 mmol/L in 2015, there were 6 children (0.5%) above this cutoff in 2016. Of the 18 children who had TC > 6.0 mmol/L in 2015, 15 were tested genetically for FH. None of them was diagnosed with FH, indicating that a TC > 6.0 mmol/L may not be a good screening parameter for FH detection in healthy children. Three children (2 of them siblings) were not tested due to parental refusal.

### Individual Variations in Lipid Levels

While the analysis of reliability showed that there is little variation in lipid levels when examined in the context of the entire population, there appears to be considerable variation at an individual level. The current study operates with 3 TC categories: healthy levels (2.5-5.2 mmol/L), hypercholesterolemia (5.2-5.9 mmol/L) and suspicion of FH (>6 mmol/L). The fluctuation between these categories is important to evaluate, especially when using TC measurements as a basis for FH screening. In the present study, we observed a higher fluctuation in TC levels in the children with the highest TC levels (variation of up to 1 mmol/L) suggesting that only TC screening may not be a good predictor for FH. Furthermore, 79 (7.5%) children changed category from 2015 to 2016 in TC levels thus suggesting that variations in TC levels occur over 1 year. This indicates that a single TC measurement may be unreliable for assessing risk of disease in children. Cholesterol might need multiple measurements to obtain reliable values, or it should be included in a broader set-up of measurements, such as in a HOMA-score. Our findings merit further studies on the biological variation, development, and fluctuations of TC levels in children across time. This includes determining the etiology of the fluctuations, and whether the fluctuation profile varies between individuals. It would also be important to investigate in longitudinal studies whether children that fluctuate up to high cholesterol levels are more at risk of high cholesterol levels in adulthood and if they show increased risk of CVD compared to individuals who fluctuate at lower cholesterol levels.

Other studies have found similar fluctuation in lipids in children. In the Bogalusa study, 196 children were classified in the highest quintile of TC at year 1. After 8 years, only 55% remained in the highest quintile.^25^ After 12 years, about 50% of the children that had a TC or LDL cholesterol levels above the 75th percentile remained elevated.^26^ The U.S. Center for Disease Control and Prevention (CDC) and American Academy of Pediatrics (AAP) agree about the possibility for decline of high cholesterol in children over time, even without intervention.^5,27^ It is also likely that some of the variation, especially a shift toward less extreme values in the second measurement, is due to a regression toward the mean. Therefore, repeated measurements are preferred over a single measurement, resulting in a higher predictive power for FH and risk of CVD.

As childhood obesity is increasing worldwide, there is an ongoing debate on whether screening of TC should be done routinely, but there is no consensus about a screening program. Some advise a family-based cascade screening program, while others recommend national lipid screening followed by a secondary genetic screening as a better approach.^28^ With increasing genome technological capability, genetic screening across all children may become a feasible strategy in the future.

### Genetic Test

For children with high TC and no genetic predisposition for FH, it is important to determine the cause of hypercholesterolemia. No evidence for decreased physical activity or overweight and obesity in this group was found. None of the 15 children that had hypercholesterolemia and who tested negative for FH were overweight or obese suggesting that the cause may be dietary.^14^ High TC has been linked to a high intake of saturated fats and specific diet predictors such as trans fatty acids, but the current study lack conclusive data diet.^29^ There may be other genetic factors connected with raised cholesterol level than those currently being tested. In Norway, there are approximately 200 mutations in the LDL receptor gene that cause FH; 4 of these are responsible for 47% of all FH. A proportion of patients with FH have an accompanying mutation that causes high fibrinogen and a greater tendency toward development of CVD. No medication is needed in FH without elevated TC and LDL below the established cut-off point, most likely caused by presence of the compensatory genes.^30^

Different reasons for elevated cholesterol occur between the healthy levels (below 5.2 mmol/L) and a highly pathological ones (over 12.0 mmol/L).^30^ In our study, all 15 children with TC over 6.0 mmol/L, without confirmed FH, were found to be in this range. The responsible mechanism for hypercholesterolemia in children in this case could be a multifactorial genetic impact with diverse effects on cholesterol.^5^ Polygenic variations of TC across the interval 5.2 to 12 mmol/L should, therefore, be investigated using more precise diagnostic technologies. Furthermore, the lack of sensitivity of a single measurement of TC > 6.0 mmol/L as a cut-off point for FH-screening calls for a re-definition of a more reliable threshold for diagnosis of FH for children. As the prevalence of FH is expected to be 1 in 200 to 300,^7^ there should have been, at least, 8 to 11 children with FH among the 2271 children measured in 2015. As none of the 15 tested for FH were diagnosed, it is likely that there are several children with FH that have a TC below 6.0 mmol/L. Thus, a single lipid measurement combined with the current threshold for FH-screening for TC in Norway is insufficient to identify FH.

### Lipid Levels

In the current study, Pearson correlation analysis of lipid changes for TC, HDL,and nonHDL showed that most of the variation in TC was explained by variation in nonHDL, with some of the variation being explained by variation in HDL. There is a weak negative correlation between nonHDL and HDL. The HDL concentration is usually less than half of LDL concentrations, thus implying that LDL (or nonHDL) has a greater impact on variability of the TC levels than HDL. Identifying factors that influence changes in nonHDL cholesterol in children is therefore important for reducing cholesterol in children that have a high TC but not related to FH.

### Limitations

Blood samples were collected in a non-fasting state; however, recent data show good validity of lipid profiles in the non-fasting state. No information was collected about ethnicity, onset of menarche or puberty in our sampled population. The current study lack data on family history of CVD and prevalence of FH among relatives.

## Conclusion

Total cholesterol values in the present study were comparable to studies in other countries. While TC and nonHDL remained the same from 2015 to 2016, HDL increased. The prevalence of hypercholesterolemia was similar in both years. Of 18 children with TC > 6.0 mmol/L in 2015, 15 were tested genetically, however none were diagnosed with FH. The prevalence of candidates qualifying for FH screening dropped from 1.3% in 2015 to 0.5% in 2016. The mean lipid values show limited annually variations, but in some children lipid values vary considerably. Changes in nonHDL cholesterol could be responsible for most of the changes in TC. The individual variation in cholesterol needs to be better understood before making recommendations about screening of cholesterol levels. A single TC measurement may not be a good predictor for FH, and there is a great need for a consensus about a screening strategy for FH.

## References

[1] Nelson RH. Hyperlipidemia as a risk factor for cardiovascular disease. Prim Care. 2013;40(1):195-211. 23402469 10.1016/j.pop.2012.11.003 PMC3572442

[2] Furtado JM Almeida SM Mascarenhas P , et al. Anthropometric features as predictors of atherogenic dyslipidemia and cardiovascular risk in a large population of school-aged children. PLoS One. 2018;13(6):e0197922. 29856786 10.1371/journal.pone.0197922 PMC5983423

[3] Johnson CO Nguyen M Roth GA Nichols E Alam T Abate D. Global, regional, and national burden of neurological disorders, 1990-2016: a systematic analysis for the global burden of disease study 2016. Lancet Neurol. 2019;18(5):459-480. 30879893 10.1016/S1474-4422(18)30499-X PMC6459001

[4] Simmonds M Burch J Llewellyn A , et al. The use of measures of obesity in childhood for predicting obesity and the development of obesity-related diseases in adulthood: a systematic review and meta-analysis. Health Technol Assess. 2015;19:1-336. 10.3310/hta19430 PMC4781104 26108433

[5] American Academy of Pediatrics, Committee on Nutrition. Cholesterol in childhood. Pediatrics. 1998;101(1 Pt 1):141-147. 11345978

[6] Raitakari OT Juonala M Kähönen M , et al. Cardiovascular risk factors in childhood and carotid artery intima-media thickness in adulthood: the cardiovascular risk in young Finns study. JAMA. 2003;290(17):2277-2283. 14600186 10.1001/jama.290.17.2277

[7] de Ferranti SD . Familial hypercholesterolemia in children and adolescents: a clinical perspective. J Clin Lipidol. 2015;9(5 Suppl):S11-S19. 10.1016/j.jacl.2015.04.009 26343208

[8] Wiegman A. Lipid screening, action, and follow-up in children and adolescents. Curr Cardiol Rep. 2018;20(9):80. 30090990 10.1007/s11886-018-1014-7 PMC6097065

[9] Langslet G Ose L. Screening methods in the diagnosis and assessment of children and adolescents with familial hypercholesterolemia. Expert Rev Cardiovasc Ther. 2013;11(8):1061-1066. 23984929 10.1586/14779072.2013.814851

[10] Svendsen K Langslet G Krogh HW , et al. Genetic testing is essential for initiating statin therapy in children with familial hypercholesterolemia: examples from Scandinavia. Atherosclerosis. 2021;316:48-52. 33302044 10.1016/j.atherosclerosis.2020.11.027

[11] Fredriksen PM Hjelle OP Mamen A Meza TJ Westerberg AC. The health oriented pedagogical project (HOPP) - a controlled longitudinal school-based physical activity intervention program. BMC Public Health. 2017;17(1):370. 28454531 10.1186/s12889-017-4282-z PMC5410047

[12] Helsebiblioteket.no. Pediatriveiledere fra Norsk barnelegeforening. 2009. Accessed March 10, 2021. https://www.helsebiblioteket.no/pediatriveiledere.

[13] Ferranti S Newburger J. Dyslipidemia in children: Definition, screening, and diagnosis - UpToDate. Updated March 3, 2020. Accessed March 4, 2021. https://www.uptodate.com/contents/dyslipidemia-in-children-definition-screening-and-diagnosis

[14] Cole TJ Bellizzi MC Flegal KM Dietz WH. Establishing a standard definition for child overweight and obesity worldwide: international survey. BMJ. 2000;320(7244):1240-1243. 10797032 10.1136/bmj.320.7244.1240 PMC27365

[15] Revelle W. Psych: Procedures for Psychological, Psychometric, and Personality Research. Northwestern University; Accessed April 2020. https://CRAN.R-project.org/package=psych

[16] Chandar V Gidvani CH Gupta AK Wilson CG Sharma YV. Lipid profile in normal healthy children. Med J Armed Forces India. 1994;50(2):101-104. 28769178 10.1016/S0377-1237(17)31008-0 PMC5529694

[17] López Martínez D Plaza Pérez I Muñoz Calvo MT , et al. [The Fuenlabrada study: lipids and lipoproteins in children and adolescents]. An Esp Pediatr. 1989;31(4):342-349. 2697162

[18] Nielsen TRH Lausten-Thomsen U Fonvig CE , et al. Dyslipidemia and reference values for fasting plasma lipid concentrations in Danish/North-European White children and adolescents. BMC Pediatr. 2017;17(1):116. 28454530 10.1186/s12887-017-0868-y PMC5410076

[19] Nguyen D Kit B Carroll M. Abnormal cholesterol among children and adolescents in the United States, 2011-2014. NCHS Data Brief. 2015;228:1-8. 26727279

[20] Wijndaele K White T Andersen LB , et al. Substituting prolonged sedentary time and cardiovascular risk in children and youth: a meta-analysis within the International Children’s accelerometry database (ICAD). Int J Behav Nutr Phys Act. 2019;16(1):96. 31672163 10.1186/s12966-019-0858-6 PMC6822444

[21] Millán J Pintó X Muñoz A , et al. Lipoprotein ratios: physiological significance and clinical usefulness in cardiovascular prevention. Vasc Health Risk Manag. 2009;5:757-765. 19774217 PMC2747394

[22] Dathan-Stumpf A Vogel M Hiemisch A , et al. Pediatric reference data of serum lipids and prevalence of dyslipidemia: results from a population-based cohort in Germany. Clin Biochem. 2016;49(10-11):740-749. 26948098 10.1016/j.clinbiochem.2016.02.010

[23] Bell MM Joseph S. Screening 1140 fifth graders for hypercholesterolemia: family history inadequate to predict results. J Am Board Fam Pract. 1990;3(4):259-263. 2248092

[24] van Vliet M Heymans MW von Rosenstiel IA Brandjes DP Beijnen JH Diamant M. Cardiometabolic risk variables in overweight and obese children: a worldwide comparison. Cardiovasc Diabetol. 2011;10(1):106. 22114790 10.1186/1475-2840-10-106 PMC3258193

[25] Freedman DS Shear CL Srinivasan SR Webber LS Berenson GS. Tracking of serum lipids and lipoproteins in children over an 8-year period: the Bogalusa heart study. Prev Med. 1985;14(2):203-216. 4048083 10.1016/0091-7435(85)90036-2

[26] Webber LS Srinivasan SR Wattigney WA Berenson GS. Tracking of serum lipids and lipoproteins from childhood to adulthood. The Bogalusa heart study. Am J Epidemiol. 1991;133(9):884-899. 2028978 10.1093/oxfordjournals.aje.a115968

[27] Moll PP Sing CF Weidman WH , et al. Total cholesterol and lipoproteins in school children: prediction of coronary heart disease in adult relatives. Circulation. 1983;67(1):127-134. 6847791 10.1161/01.cir.67.1.127

[28] Klančar G Grošelj U Kovač J , et al. Universal screening for familial hypercholesterolemia in children. J Am Coll Cardiol. 2015;66(11):1250-1257. 26361156 10.1016/j.jacc.2015.07.017

[29] Siri-Tarino PW Sun Q Hu FB Krauss RM. Saturated fatty acids and risk of coronary heart disease: modulation by replacement nutrients. Curr Atheroscler Rep. 2010;12(6):384-390. 20711693 10.1007/s11883-010-0131-6 PMC2943062

[30] Wiegman A Gidding SS Watts GF , et al. Familial hypercholesterolaemia in children and adolescents: gaining decades of life by optimizing detection and treatment. Eur Heart J. 2015;36(36):2425-2437. 26009596 10.1093/eurheartj/ehv157 PMC4576143

